# Disentangling Gut Microbiome Alterations in Children with Cow’s Milk Allergy: Impact of Sex, Milk Elimination, and Family History of Allergies

**DOI:** 10.3390/nu18030398

**Published:** 2026-01-26

**Authors:** E. Daniel León, Dafni Moriki, Alejandro Artacho, Xavier Pons, Despoina Koumpagioti, Sophia Tsabouri, Kostas N. Priftis, Konstantinos Douros, M. Pilar Francino

**Affiliations:** 1 Department of Genomics and Health, Fundació per al Foment de la Investigació Sanitària I Biomèdica de la Comunitat Valenciana (Fisabio), 46020 Valencia, Spain; daniel.leon@fisabio.es (E.D.L.); alejandro.artacho@fisabio.es (A.A.); javier.pons@fisabio.es (X.P.); 2 3rd Department of Pediatrics, School of Medicine, National and Kapodistrian University of Athens, “Attikon” University Hospital, 12462 Athens, Greece; dafnimoriki@yahoo.gr (D.M.); kpriftis@otenet.gr (K.N.P.); costasdouros@gmail.com (K.D.); 3 Department of Nursing, University of West Attica, 12243 Athens, Greece; dkoumpagioti@uniwa.gr; 4 Department of Pediatrics, University Hospital of Ioannina, 45500 Ioannina, Greece; stsabouri@gmail.com; 5 CIBER en Epidemiología y Salud Pública, 28029 Madrid, Spain

**Keywords:** gut microbiome, cow’s milk allergy (CMA), development of oral tolerance (DOT), milk elimination, family history of allergy, school-age children, sex-linked microbiome differences, *Monoglobus*, *Anaerostipes*

## Abstract

**Background**: Children with cow’s milk allergy (CMA) present alterations in their gut microbiome, but any potential sex-dependency of these has not been addressed. Further, whether eliminating milk from children’s diet has similar effects on the gut microbiomes of boys and girls is also not known. Here, our main objective is to analyze how CMA and development of oral tolerance (DOT) to milk proteins affect the gut microbiota in female and male children. We also perform exploratory analyses to investigate whether milk elimination and/or a family history of allergies underlie sex-associated differences. **Methods**: We obtained 16S rRNA gene sequences of the intestinal microbiota of 32 children aged 5–12 years with CMA, of which 14 had active CMA and 18 had developed oral tolerance, along with 36 age-matched healthy controls (51.5% male). PERMANOVA and differential abundance analyses were employed to evaluate overall compositional differences and to identify bacteria varying between the groups. **Results**: The effects of CMA on the gut microbiome are more pronounced in girls, including female-specific decreases in bacteria potentially related to protection from allergy, such as *Monoglobus* and *Anaerostipes*. The girls’ microbiomes were also found to be more influenced by a family history of allergy, remaining farther from the healthy state upon DOT. In contrast, milk elimination affects more taxa in boys in the control group than in girls in the control group, although it alters global microbiome composition in both. In all, milk elimination and family history fail to explain most microbiome alterations observed in CMA, indicating that the latter are specifically linked to disease development. **Conclusions**: Gut microbiome alterations associated with CMA are sex-dependent, suggesting that sex-specific strategies, dietary and otherwise, may be more effective at modulating them toward healthier states.

## 1. Introduction

The gastrointestinal tract is the habitat of the largest population of commensal bacteria in the human body. This bacterial community plays an important role in health and disease, and has been implicated in metabolic, inflammatory, neurodegenerative, and atopic diseases, including food allergies [1,2,3,4,5]. In particular, several works [6,7,8,9,10] have described a relation between the gut microbiota and cow’s milk allergy (CMA), the most common food allergy in childhood, caused by abnormal immune responses against the proteins found in cow’s milk. Although most children undergo the development of oral tolerance (DOT) by four years of age [11], CMA can persist in older children and even into adolescence and adulthood, resulting in long-term dietary restrictions that affect nutrition, health, and quality of life [12].

To date, there is no effective treatment for CMA other than avoiding milk consumption and seeking emergency treatment in cases of accidental exposure [13]. However, milk elimination does not stop the inflammation triggered by CMA [14]. Therefore, novel preventive and therapeutic strategies are clearly required, and microbiome-based approaches are amongst the most promising. Studies in animal models suggest that colonization with certain bacteria may protect against food allergies. *Bifidobacterium* and certain species of *Clostridium* can be protective through their direct induction of mucosal regulatory T cells (Tregs) and the improvement of gut permeability, which reduces food allergen sensitization [15,16]. In addition, several members of the Lachnospiraceae family have also been shown to protect against food allergies by reinforcing the epithelial barrier and reducing gut permeability to dietary proteins through the stimulation of IL-22 production [17]. In mouse models, the administration of a synbiotic combining the Lachnospiraceae species *Anaerostipes caccae* with the prebiotic lactulose succeeded in preventing and treating CMA [18], possibly aided by a high production of the anti-inflammatory short-chain fatty acid butyrate, which induces mucosal and peripheral Tregs [19]. Although such results in experimental models are highly encouraging, a better understanding of the relation between commensal gut bacteria, milk elimination, and CMA in humans should help generate the best microbiome-based strategies for disease management.

Furthermore, intestinal microbiota alterations in relation to both disease and diet may be influenced by sex. Gut microbiota composition has been shown to depend on interactions between diet and sex in populations of wild and laboratory fish, laboratory mice, and humans [20], and is likely related to sex hormones and sex differences in immune function. Also, sex-specific gut microbiome dysbioses have been reported for a variety of diseases in adult humans, including obesity [21], metabolic syndrome [22], cardiovascular diseases [23], irritable bowel syndrome [24], and depression [25], and a recent report suggests different microbiome alterations in boys and girls with autism [26]. The origin and relevance of these sex-specific associations between gut microbiota and disease are not understood, but immune differences between males and females are likely implicated. Important sex-associated differences in innate and adaptive immunity have been described in both children and adults: males and females differ in their immune responses to both foreign and self-antigens, with females typically exhibiting stronger responses to vaccines and microbial infections, as well as having a higher propensity towards autoimmune disorders [27,28]. Given the strong interaction between immunity and the intestinal microbiota, such differences could direct distinct microbiota changes in males and females, especially during immune disease. Sex-specific microbiome differences associated with distinct immune responses have been demonstrated in a mice model for an autoimmune disease such as type 1 diabetes [29].

In the case of CMA, sex-associated epigenetic differences have been identified, with methylation alterations affecting different genes in each sex. This suggests that different biological mechanisms may underlie CMA in boys and girls [30]. Epidemiological data also indicate differences in CMA prevalence and prognostics between sexes [31]. However, the extent to which sex-associated differences occur in the intestinal microbiota in CMA has not been previously explored.

In this work, we examined whether a sex-specific signature exists in the gut microbiota of children with CMA, including those that have developed oral tolerance (DOT) to milk proteins. As the gut microbiota may respond differently to diet alterations in males and females, we also examined whether milk elimination has differential effects in boys and girls. Finally, we evaluated how the presence of a family history of allergies affects the children’s microbiota, and whether it does so differently according to sex. In all, our analyses allow us to investigate whether sex-associated differences in the gut microbiota of CMA children derive from diet–sex interactions or whether they are more likely driven by non-dietary factors.

## 2. Materials and Methods

### 2.1. Study Population

Sixty-eight children were recruited at the Pediatric Allergy and Respiratory Unit of the “Attikon” University Hospital in Athens from January 2021 to December 2023, as described by Moriki et al. [10]. Briefly, the study involved 32 children aged 5–12 years having a history of CMA, of which 14 had active CMA and 18 had developed oral tolerance (DOT), along with 36 age-matched controls without CMA. Both the control and the DOT groups included children that did or did not consume milk. The mean age of the children was 7.3 (2.1) (standard deviation, (SD)) years, and 35 (51.5%) were boys. Relevant clinical and dietary variables considered in this study and the number of children per group are presented in Table 1.

The diagnosis of CMA was based on detection of specific Immunoglobulin (Ig) E antibodies against cow’s milk, medical history, and oral food challenge tests according to the guidelines of the European Academy of Allergology and Clinical Immunology [32], the American Academy of Allergy, Asthma, and Immunology [33], and the National Institute for Health and Clinical Excellence [34,35]. A detailed medical history including information on any family history of allergic diseases was provided by the children’s parents.

Exclusion criteria comprised the following: presence of other food allergies without development of oral tolerance, presence of chronic gastrointestinal disorders and/or other severe chronic diseases, and consumption of probiotics, corticosteroids, antibiotics, and other medications that affect the gut microbiome within the three months before the study. Written informed consent was obtained from the parents of all participants. The study was approved by the Ethics Committee of the University General Hospital “Attikon” (546/1-10-20).

### 2.2. Gut Microbiota Composition

This work analyzed 16S rRNA gene sequencing data obtained previously by our team [10] (European Nucleotide Archive accession number PRJEB78556).

Briefly, stool samples were collected during study visits or by the parents at home using a stool collection kit and transported to the research center within 24 h. Then, 10 g of feces were placed in a sterile 50 mL Falcon tube containing 10 mL of RNA later solution and frozen at −80 °C. All samples were sent to FISABIO (Valencia, Spain) in Styrofoam boxes with dry ice for analysis. Bacterial pellets from 50 mL Falcon tubes were lysed using 0.1 mg/mL lysozyme at 37 °C for 30 min. The MagNaPure LC JE379 platform and DNA Isolation Kit III were used for DNA extraction. DNA was quantified with a Qubit 3.0 Fluorometer (Invitrogen, Waltham, MA, USA) and stored at −20 °C. The V3-V4 hypervariable region of the 16S rRNA gene was amplified using 12 ng of DNA. PCR was performed with forward primer 5′-TCGT CGGC AGCG TCAG ATGT GTAT AAGA GACA GCCT ACGG GNGG CWGC AG-3′ and reverse primer 5′-GTCT CGTG GGCT CGGA GATG TGTA TAAG AGAC AGGA CTAC HVGG GTAT CTAA TCC-3′, with adapter sequences for compatibility with the Illumina Nextera XT Index kit. Amplicon libraries were pooled and sequenced in an Illumina Miseq sequencer in paired-end runs of 2 × 300 cycles (MiSeq Reagent kit v3, Illumina, San Diego, CA, USA).

The DADA2 (v1.8.0) package in R (v3.6.0) was used for sequence read processing, forward and reverse read merging, and clustering into amplicon sequence variants (ASVs) [36]. Filtering and trimming parameters were as follows: maxN = 0, maxEE = c (2, 5), truncQ = 0, trimLeft = c (17, 21), truncLen = c (270, 220), and rm.phix = TRUE. Specifications for merging the reads were a minimum overlap of 15 nucleotides and a maximum mismatch of 1. Taxonomic identification was assigned to ASVs using DADA2 and the SILVA v.138 reference database.

### 2.3. Statistical Analysis

The Vegan package (v2.5-2) on the R platform was used to analyze beta diversity in gut microbiota composition, i.e., differences in composition between samples. The Bray–Curtis dissimilarity index was applied to quantify the overall measure of dissimilarity between two microbial communities and was used in permutational multivariate analysis of variance (PERMANOVA) to evaluate overall compositional differences among population groups. The Adonis function included in the Vegan package was employed to perform PERMANOVA with 600 permutations at ASV level. Analysis of the composition of microbiomes (ANCOM-BC2) [37] was used to identify differentially abundant taxa among samples from different groups. A table normalized for compositional biases in microbiome data was obtained from ANCOM-BC2 and a Wilcoxon rank–sum test was performed to evaluate the significance of abundance differences. The Benjamini–Hochberg procedure was used for false discovery rate (FDR) control as described in Kaul et al. [38] and results are presented for taxon comparisons yielding FDR-adjusted *p* values <0.15 (*), <0.10 (**) and <0.05 (***).

## 3. Results

### 3.1. The Gut Microbiota of Boys and Girls Is Affected Differently by Both CMA and DOT

We first established whether differences in gut microbiota composition could be detected between all boys and girls in our study population; when we compared all the female children (F) with all the male children (M), we did not detect a difference (*p* = 0.64). Then, we compared F vs. M within the groups of HEALTHY (control), CMA, and DOT children. These comparisons revealed that, while gut microbiota composition did still not differ between F.HEALTHY and M.HEALTHY (*p* = 0.47), differences were close to significance for F.CMA vs. M.CMA (*p* = 0.055) and for F.DOT vs. M.DOT (*p* = 0.056).

**Figure 1:**
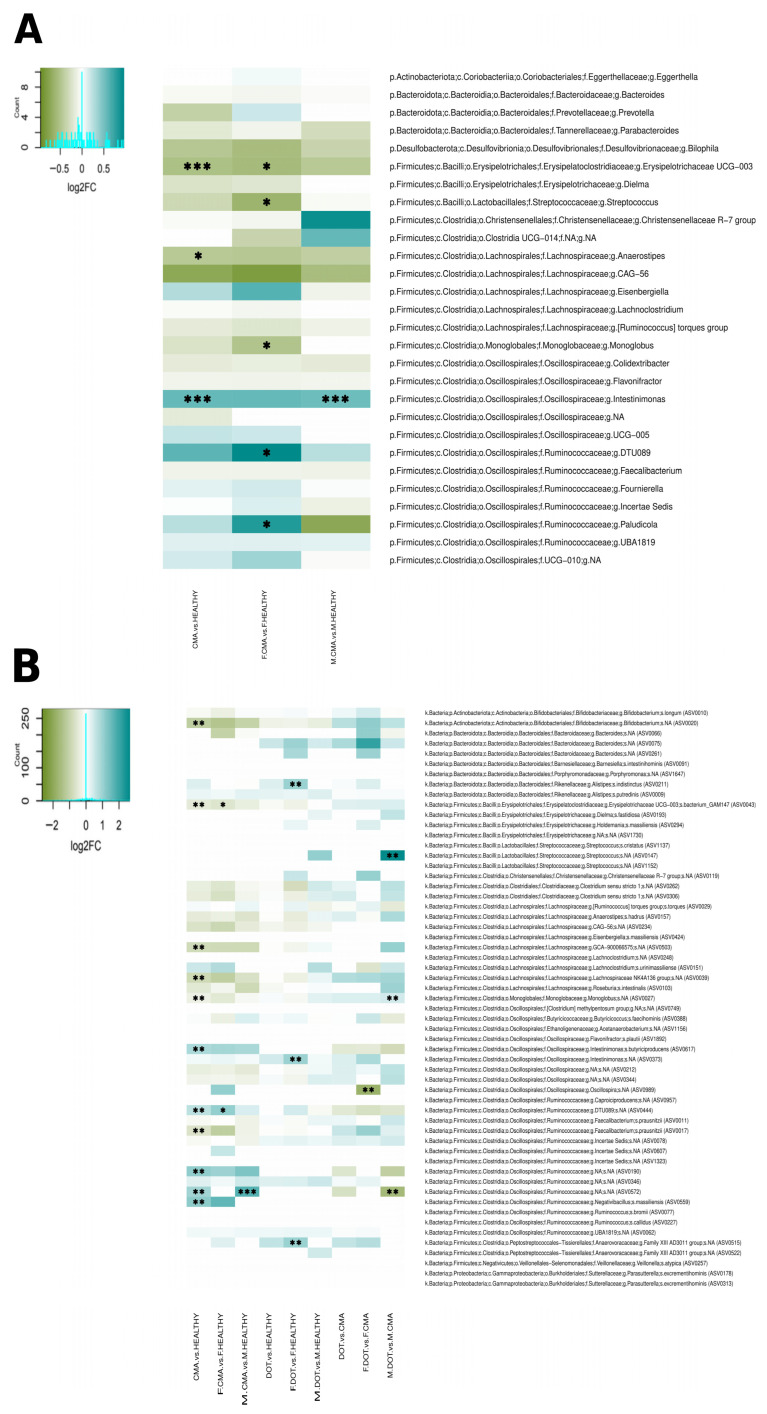
Gut microbiota alterations in CMA and DOT in unstratified and sex-stratified analyses (F and M). Differentially abundant taxa were identified through Wilcoxon rank–sum tests performed on abundance data following ANCOM-BC2 normalization, for CMA vs. HEALTHY, DOT vs. HEALTHY and DOT vs. CMA comparisons. Heatmaps of log2 fold change (log2FC) values for bacterial relative abundances are presented at genus (**A**) and ASV (**B**) levels. Comparisons yielding FDR-adjusted *p* values <0.15 (*), <0.10 (**) and <0.05 (***) are indicated.

We then investigated the potential differences in the effects of CMA on the gut microbiota of boys and girls by analyzing health categories separately for each sex and asking whether the level and type of differences were the same in both sexes. In the CMA vs. HEALTHY comparison, irrespective of sex, there was a significant difference in the microbiota composition of the two groups (*p* = 0.002), in accordance with previous results by us [10] (Moriki et al., 2024) and others. The *Intestinimonas* genus as well as several ASVs belonging to *Intestinimonas butyriciproducens*, *Negativibacillus massiliensis,* and various Ruminococcaceae were over-represented in CMA. On the other hand, the Erysipelotrichaceae UCG-003 group and the genus *Anaerostipes*, as well as ASVs belonging to Erysipelotrichaceae UCG-003, *Monoglobus*, *Faecalibacterium prausnitzii*, Lachnospiraceae NK4A136, Lachnospiraceae GCA-900066575, and *Bifidobacterium*, were under-represented (Figure 1A,B). However, when we separately compared F.CMA vs. F.HEALTHY and M.CMA vs. M.HEALTHY, the comparison in girls resulted in a highly significant difference (*p* = 0.002), whereas in boys the significance was less pronounced (*p* = 0.041). Moreover, different bacteria were differentially abundant between CMA and healthy children in boys and girls. In girls, *Paludicola* and Ruminococcaceae DTU089 were over-represented in CMA, while Erysipelotrichaceae UCG-003, *Monoglobus*, and *Streptococcus* were under-represented. In contrast, only *Intestinimonas* and an ASV of the family Ruminococcaceae increased in boys with CMA (Figure 1A,B).

To understand potential sex differences in microbiota change during the development of oral tolerance to milk proteins, we analyzed DOT vs. HEALTHY and DOT vs. CMA. The DOT vs. HEALTHY comparisons revealed no significant differences in overall microbiota composition for boys and girls together (*p* = 0.27) or for boys alone (*p* = 0.13), while significant differences were detected for girls (*p* = 0.017), with increases in DOT of ASVs belonging to *Intestinimonas*, *Alistipes indistinctus,* and the Anaerovoracaceae family (Figure 1B). On the contrary, DOT vs. CMA revealed a moderate difference for boys and girls together (*p* = 0.044), a highly significant difference for boys (*p* = 0.001) and no significant difference for girls (*p* = 0.12). In boys, an ASV of the Ruminococcaceae family that was increased in CMA decreased in DOT (ASV0572), while *Monoglobus* and *Streptococcus* ASVs increased; in contrast, only a single ASV belonging to *Oscillospira* decreased in DOT for girls (Figure 1B). This indicates that overall gut microbiota composition in DOT changes towards that of HEALTHY children in boys, whereas in girls it remains similar to that in CMA.

### 3.2. Milk Elimination Affects the Gut Microbiome Composition of Both Boys and Girls but Alters the Abundances of Different Bacteria

**Figure 2:**
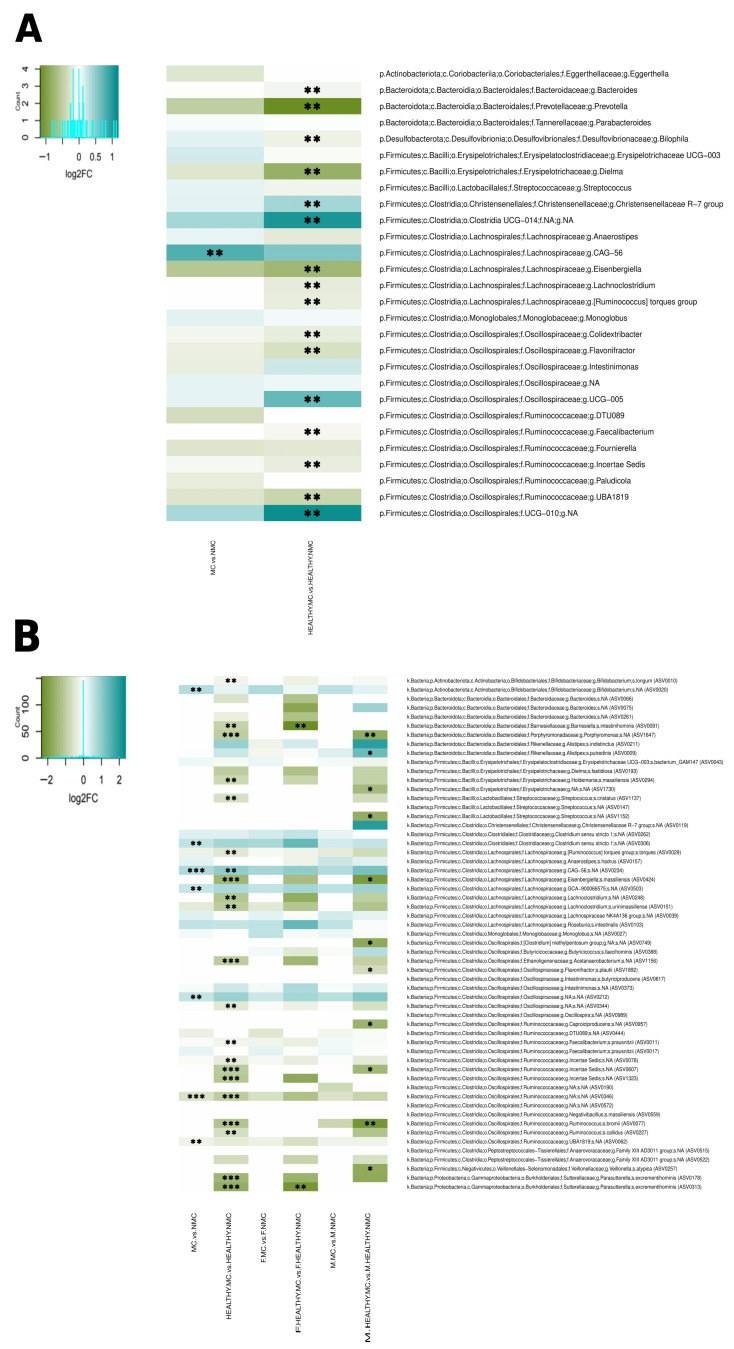
Impact of milk elimination on the gut microbiota in unstratified and sex-stratified analyses (F and M) for all children and children in the control group only (HEALTHY). Differentially abundant taxa were identified through Wilcoxon rank–sum tests performed on abundance data following ANCOM-BC2 normalization, for MC vs. NMC and HEALTHY.MC vs. HEALTHY.NMC comparisons. Heatmaps of log2 fold change (log2FC) values for bacterial relative abundances are presented at genus (**A**) and ASV (**B**) levels. Comparisons yielding FDR-adjusted *p* values <0.15 (*), <0.10 (**) and <0.05 (***) are indicated. MC = milk consumers; NMC = non-milk consumers.

Milk consumption has a significant effect on the gut microbiota composition of children. When all milk consumers in our population were compared to non-milk consumers (irrespective of sex and allergic status) a significant difference was identified in overall microbiota composition (*p* = 0.01). Non-consumers had a decreased abundance of the Lachnospiraceae CAG-56 genus-level group and of ASVs belonging to this group (ASV0234), Lachnospiraceae GCA-900066575, *Clostridium sensu stricto* 1, Oscillospiraceae, and *Bifidobacterium*, as well as an over-representation of two ASVs belonging to the Ruminococcaceae (ASV0346 and ASV0062) (Figure 2A,B). This difference is not due exclusively to the presence of the allergic children among the non-milk consumers, as a very significant difference is also detected when only healthy milk consumers and non-milk consumers are compared (*p* = 0.02). Healthy non-consumers presented decreased abundances of the Lachnospiraceae CAG-56 ASV0234 and of various genus-level groups of the Clostridia class. In addition, they also presented over-representations of a large variety of genus-level groups and ASVs, mostly belonging to the families Ruminococcaceae, Lachnospiraceae, and Oscillospiraceae, as well as the order Bacteroidales (Figure 2A,B).

When we compare milk consumers to non-milk consumers with stratification by sex, overall compositional differences are modest in both boys (*p* = 0.031) and girls (*p* = 0.078) when all children (healthy and non-healthy) are included, with no specific bacteria identified in either sex as differentially abundant. In contrast, very significant overall compositional differences (*p* = 0.001) are evidenced in both sexes when only the children in the control group are considered. However, differential abundance analyses reveal different patterns in boys and girls, with many more differentially abundant bacteria detected in boys. Boys and girls in the control group who did not consume milk presented increased abundances of different sets of bacteria among those detected in the unstratified comparison, i.e., ASVs in the Ruminococcaceae, Lachnospiraceae, and Bacteroidales (*Porphyromonas*) in the case of boys, and in the Sutterellaceae and Bacteroidales (*Barnesiella*) in the case of girls. In addition, non-consumer boys in the control group also presented increased abundances of ASVs belonging to Clostridia and other Bacillota classes, as well as a decreased abundance of an ASV belonging to *Alistipes putredinis* (Figure 2B).

### 3.3. Having a Family History of Allergies Has a Stronger Impact on the Gut Microbiota in Girls

**Figure 3:**
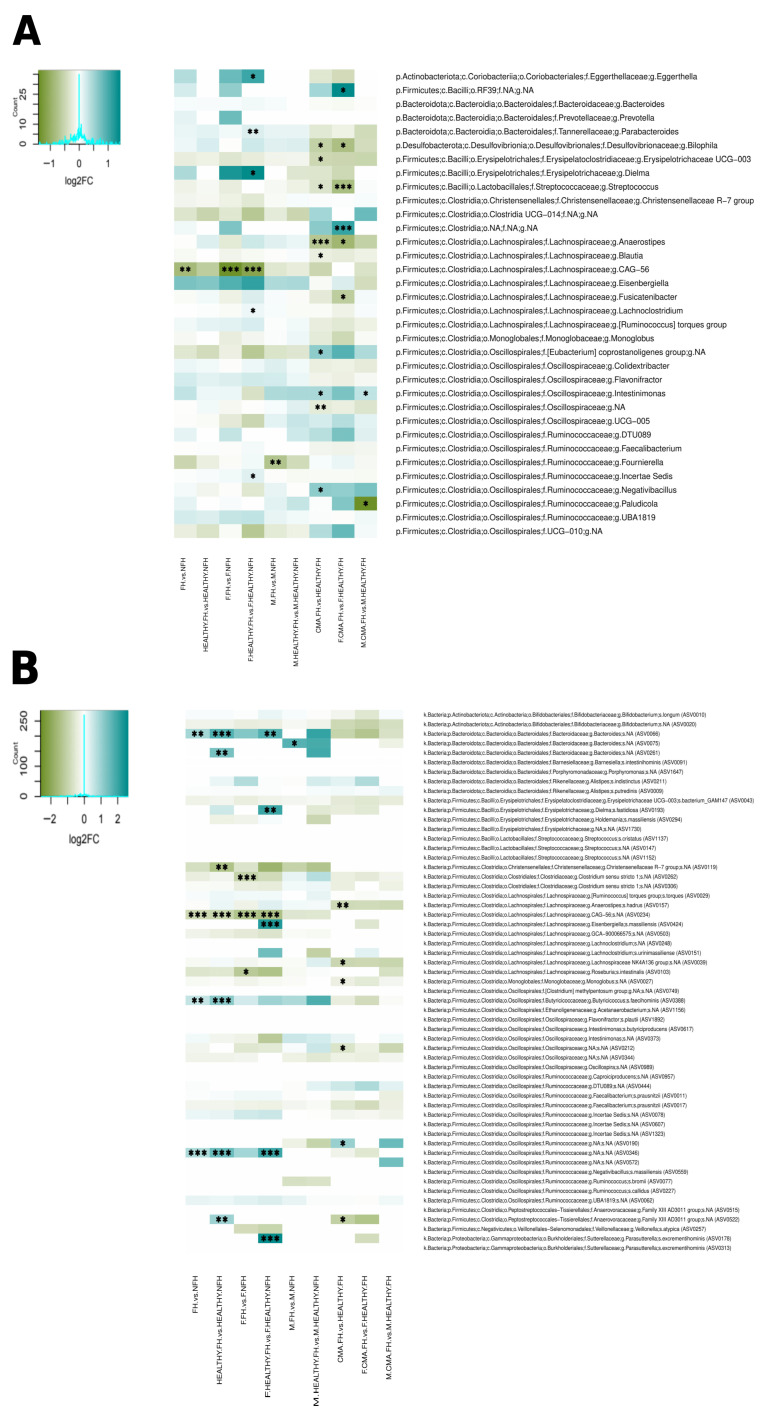
Gut microbiota alterations associated with having a family history of allergic disease (for all children and children in the control group only) and differences between children with CMA and children in the control group when a family history is present. In each case, unstratified and sex-stratified analyses (F and M) are presented. Differentially abundant taxa were identified through Wilcoxon rank–sum tests performed on abundance data following ANCOM-BC2 normalization, for FH vs. NFH, HEALTHY.FH vs. HEALTHY.NFH, and CMA.FH vs. HEALTHY.FH comparisons. Heatmaps of log2 fold change (log2FC) values for bacterial relative abundances are presented at genus (**A**) and ASV (**B**) levels. Comparisons yielding FDR-adjusted *p* values <0.15 (*), <0.10 (**) and <0.05 (***) are indicated. FH = family history, NFH = no family history.

Comparing all children with a family history (FH) of allergies vs. those having no family history (NFH) revealed significant differences between the two groups (*p* = 0.03). Like non-milk consumers, FH children showed a decreased abundance of the genus-level group Lachnospiraceae CAG-56 (and its ASV0234) and an increased abundance of Ruminococcaceae ASV0346; in addition, *Bacteroides* ASV0066 and *Butyricicoccus faecihominis* ASV0388 were also elevated in this group (Figure 3A,B). Again, this difference is not due exclusively to the higher proportion of allergic children in FH, as an even more significant difference is detected when only healthy children with or without a family history of allergies are compared (*p* = 0.009). In this case, in addition to the differences involving ASV0234, ASV0346, ASV0066, and ASV0388, FH in the control group also had over-representations of two ASVs belonging to *Bacteroides* and the Anaerovoracaceae family, as well as under-representation of an ASV belonging to the Christensenellaceae (Figure 3B).

However, when a stratification by sex is included, family history of allergies only significantly affects the gut microbiota in girls (*p* = 0.024 for all and *p* = 0.001 for healthy only), but not in boys (*p* = 0.092 for all and *p* = 0.17 for healthy only). When girls with FH are considered, the decreased abundances of the genus-level group Lachnospiraceae CAG-56 and its ASV0234 are apparent, as well as decreased abundances of ASVs belonging to *Clostridium sensu stricto* 1 and *Roseburia intestinalis* that were not present in the unstratified analyses. If the analysis is restricted to girls in the control group, many of the differences detected in the unstratified analyses (involving Lachnospiraceae CAG-56, ASV0234, ASV0346 and ASV0066) are apparent; in addition, FH girls in the control group also had over-representations of various other genera and ASVs belonging to a variety of phyla (Bacillota, Bacteroidota, Actinomycetota and Pseudomonadota). In contrast, only *Fournierella* and a *Bacteroides* ASV were under and over-represented, respectively, in FH boys when all were considered, and the differences did not remain when the analysis was restricted to boys in the control group (Figure 3A,B). These results indicate that, unlike milk elimination, having a family history of allergies affects more strongly the gut microbiota of girls, even when CMA does not develop.

### 3.4. The Gut Microbiota Is More Altered by CMA in Girls than in Boys Having a Family History of Allergy

We finally looked at differences between children with FH who develop CMA (CMA.FH) and children with FH who do not develop CMA (HEALTHY.FH). These groups showed a significantly different overall microbiota composition when both boys and girls were considered (*p* = 0.005), with children who do develop CMA presenting increases in *Intestinimonas*, *Negativibacillus*, the *Eubacterium coprostanoligenes* group and Ruminococcaceae ASV0190, as well as decreases in *Bilophila* and a variety of genus-level groups and ASVs belonging to different Bacillota families (including *Streptococcus,* Erysipelotrichaceae UCG-003, *Blautia*, *Anaerostipes* and ASVs of Lachnospiraceae NK4A136 and *Monoglobus*, among others) (Figure 3A,B).

After stratification by sex, gut microbiota composition differed significantly according to whether CMA was present for both girls and boys with FH, but the difference was more pronounced in girls (girls *p* = 0.003, boys *p* = 0.020), with different bacteria detected as differentially abundant in each comparison. In girls, CMA was associated with increases in unassigned Clostridia and Bacilli, as well as decreases in *Bilophila*, *Streptococcus, Anaerostipes,* and *Fusicatenibacter*; in boys, it was only associated with an increase in *Intestinimonas* and a decrease in *Paludicola* (Figure 3A,B).

**Figure 4:**
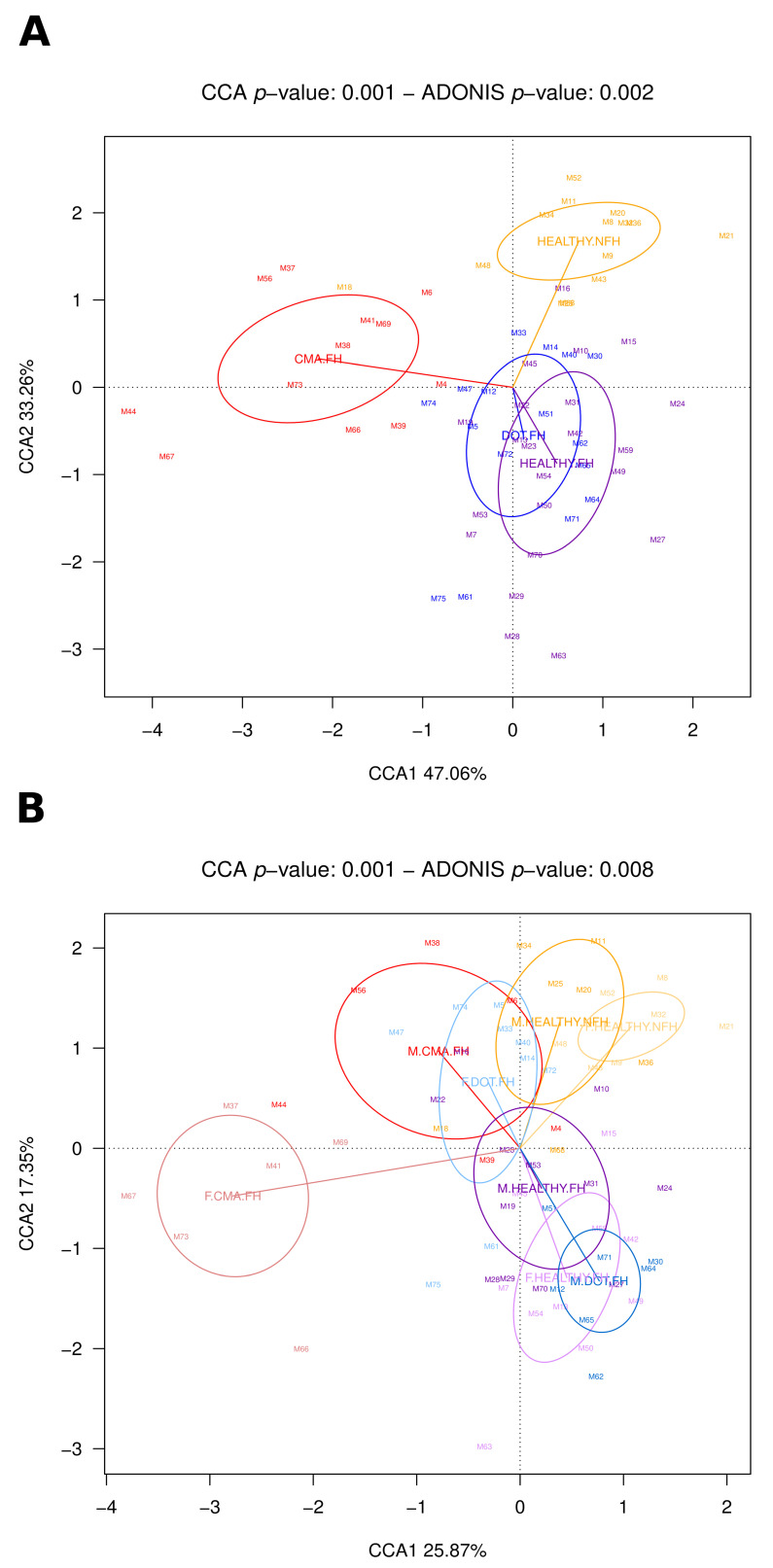
Canonical Correspondence Analysis (CCA) plots showing differentiation in overall gut microbiota composition among HEALTHY, CMA, and DOT children with FH and HEALTHY children with NFH, for boys and girls together (**A**) and after stratification by sex (**B**). In each case, overall compositional differences among all included population groups were evaluated at ASV level through PERMANOVA analyses using the Adonis function of the Vegan package in R.

We also analyzed differences in DOT vs. HEALTHY and DOT vs. CMA in children with a family history of allergies. The CCA in Figure 4A shows the localization of microbiota composition clusters corresponding to HEALTHY, CMA, and DOT children with FH for boys and girls together, with HEALTHY children with NFH included for comparison (*p* = 0.001). The first CCA axis clearly separates all three children’s groups with FH from HEALTHY.NFH, while the second axis shows a large separation between CMA.FH and HEALTHY.FH, with DOT.FH clustering between the two. After stratification by sex, the CCA (Figure 4B) shows large separations between boys and girls for both CMA.FH and DOT.FH, with CMA.FH girls located further from healthy children clusters, DOT.FH boys clustering close to HEALTHY.FH and DOT.FH girls remaining close to CMA.FH. Furthermore, although DOT.FH girls clearly cluster separately from CMA.FH girls, their cluster overlaps with that of CMA.FH boys. This reinforces the notion that DOT results in a reversion of CMA-associated gut microbiota alterations in boys, while the gut microbiota of DOT girls still presents a CMA-associated taxonomic composition.

## 4. Discussion

Our analyses of how CMA affects the gut microbiota in female and male school-age children show that CMA is associated with a more disruptive alteration in the gut microbiota of girls. It is important to note that stratification by sex reduced the sample size available for our analyses; therefore, it will be important to further investigate this issue in larger population groups. Nevertheless, in spite of the sample size reduction, our results show a clear tendency, revealing a larger difference in overall gut microbiota composition in girls with CMA (Figure 4B), as well as different alterations of specific bacterial abundances in each sex (Figure 1A,B). Girls with CMA displayed decreased abundances of bacteria (Figure 1A) potentially related to protection from allergies that were not detected in boys. Among these, the decrease in *Monoglobus* has also been detected in children with peanut allergies [39]. This reduction may be linked to the increased levels of inflammation in CMA [14], as *Monoglobus* has been negatively associated with intestinal inflammation [40] and is known to produce bile acid metabolites that inhibit the differentiation of Th17 cells and increase that of Tregs, enhancing the integrity of the intestinal barrier [41]. A negative correlation has also been reported between *Monoglobus* and plasma TNF-α, one of the main proinflammatory cytokines, and with the expression of the *TNF-α* gene in the colon, whereas a positive correlation was detected with colon expression of the tight junction genes *OCLN* and *CLDN1* [42]. These functional capacities and detected associations suggest that *Monoglobus* may be disfavored in an inflammatory environment, with its decrease promoting further inflammation. Moreover, mucosal immune activation and expression of inflammation-related genes have been shown to be higher in females [43], potentially contributing to explain why *Monoglobus* does not decrease in boys.

On the other hand, a decreased abundance of Erysipelotrichaceae has been shown to correlate with an increased expression of gene pathways related to extracellular matrix remodeling in the intestinal mucosa [44] and could, therefore, also affect the condition of the gut barrier. Moreover, this family has also been detected to decrease in pediatric inflammatory bowel disease [45] as well as upon oral allergen sensitization in a mouse model of food allergy [46], although it also displays negative associations with health, as it has been found to increase in metabolic disorders and colorectal cancer [47,48].

Increases in members of the Ruminococcaceae family were detected in both girls and boys, but different organisms were involved in each case, with the genus-level group Ruminococcaceae DTU089 increasing in girls and a single Ruminococcaceae ASV (ASV0572) increasing in boys. This family has often been implicated in food [7,49] and other allergies [50], but different Ruminococcaceae members likely have very different potential implications for disease. In addition, the Oscillospiraceae genus *Intestinimonas* was only significantly increased in boys with CMA, although it also had a high positive log2 fold change in girls, despite not reaching our criteria for significance (Figure 1). *Intestinimonas*, although frequently associated with positive health outcomes, has also been recently linked to some inflammatory conditions, such as obesity [51] and type 2 diabetes [52], and was positively associated with levels of Th2-related factors in a mouse model of allergic asthma [53].

Our results further indicate that very different gut microbiota composition changes have occurred in girls and boys who have developed tolerance (Figure 1 and Figure 4). Remarkably, microbiota composition in DOT has changed towards that of healthy children for boys, but not for girls. Progression towards a health-associated microbiome in boys is in agreement with the results from epigenetic studies showing that CMA-associated alterations of gene expression disappear with DOT in boys [30]; unfortunately, this study did not include DOT girls to evaluate whether this would also be the case in this group. In contrast, our results imply that CMA has a long-term impact on the gut microbiota of girls, even after the allergy is no longer active; therefore, these microbiota alterations could still have downstream effects on their immunity or metabolism. As mentioned above, females have higher levels of mucosal immune activation and inflammation even in the absence of pathology [43]; therefore, they may be less able to recover from the deleterious effects on the intestinal environment associated with CMA. Also, this difference reinforces the notion that immune tolerance acquisition pathways may differ between boys and girls, as the gut microbiome has been implicated in their deployment [54].

In order to investigate potential sources for the sex-associated microbiota differences in CMA, we asked whether milk elimination or having a family history of allergy could have a differential impact on boys and girls. Although these stratifications further reduced our sample size, our results revealed that none of the sex-linked alterations in the abundances of specific taxa in CMA were apparent in comparisons between milk consumers and non-consumers (Figure 2) or between children with or without a family history of allergy (Figure 3). This indicates that sex-linked microbiome differences in CMA are uniquely associated with the presence of the disease. Nevertheless, both factors did have significant impacts on the gut microbiota, even when analyses were restricted to children without CMA. Moreover, in both cases, differences were apparent between boys and girls. However, analyses with larger population sizes will be required to further elucidate sex-linked effects of these factors on the gut microbiome.

Milk elimination had a considerable impact on the gut microbiota of both sexes. However, its effects explained only some of the altered bacterial abundances observed in unstratified CMA analyses, and none of those that were sex-specific. In particular, the same *Bifidobacterium* and Lachnospiraceae GCA-900066575 ASVs were under-represented in the unstratified comparisons in both CMA (Figure 1B) and non-milk consumers (Figure 2B), suggesting that their decrease in CMA is indeed associated with the lack of milk consumption. In contrast, other bacteria strongly affected by milk consumption did not present altered abundances in CMA. This is the case for several members of the Ruminococcaceae family that were over-represented in healthy non-milk consumers, including a Ruminococcaceae *incertae sedis* and numerous ASVs. Although some Ruminococcaceae did increase in CMA (Figure 1), these were different genus-level groups or ASVs, suggesting that these members of the family are favored under specific conditions involved in CMA beyond the lack of milk consumption. Similarly, Lachnospiraceae CAG-56 (and its ASV0234) were strongly under-represented in healthy non-milk consumers, but not in CMA, where other members of the Lachnospiraceae were decreased, further supporting the different impacts of CMA and simple milk elimination on the gut environment. In regard to sex-specific differences, unlike CMA, milk elimination had a stronger impact in boys in terms of a higher number of differentially abundant taxa. Six ASVs belonging to different families of the classes Bacilli, Clostridia, Erysipelotrichia, and Negativicutes were over-represented only in non-consumer boys, but these did not include the bacteria over-represented in boys with CMA. Therefore, the observed sex-specific effects of eliminating milk from the diet cannot explain the stronger overall impact of CMA in girls or the different alterations in bacterial abundances associated with CMA in each sex.

In contrast to milk elimination, having a family history of allergy (FH) only had a significant impact on the gut microbiota of girls. Girls in the control group with FH had increased abundances of the genera *Parabacteroides* and *Dielma*, as well as of ASVs of *Dielma fastidiosa*, *Eisenbergiella massiliensis*, and *Parasutterella excrementihominis*, which were not detected in FH boys. These taxa have been reported to display several negative associations with health. Experiments in mouse models have linked *Parabacteroides* with the development of food allergies, including peanut [55], hazelnut [56], and gluten [57], and its abundance is increased in patients with eczema [58] and allergic rhinitis [59]. Importantly in our context, Xie et al. [57] showed that increases in *Parabacteroides*, along with other genera, preceded the onset of allergy, and were linked to further exacerbations of the allergic phenotype; this suggests a causal role for this taxon in the inflammation and overall alteration of intestinal homeostasis associated with the development of gluten allergy. *Parasutterella excrementihominis* has been associated with health outcomes linked with inflammatory patterns, such as inflammatory bowel disease, irritable bowel syndrome, diabetes, and fatty liver disease [60,61]. Finally, both *Dielma* and *E. massiliensis* have been associated with unhealthy dietary patterns [62,63,64] and *Dielma* has also been linked to negative health outcomes including obesity [62] and fetal growth restriction [65]. Overall, this suggests that these bacteria over-represented in FH girls in the control group may be favored by an inflammatory intestinal milieu, which may be present in children having a genetic predisposition to allergy. Genes linked to allergy risk encode proteins involved in bacterial recognition, inflammation, and regulation of the immune response; therefore, they may clearly affect the conditions of the intestinal environment and the bacteria that they select for. Moreover, the effects of mutations in these genes may be exacerbated in females, given their higher expression of inflammation-related genes, stronger responses to antigens and augmented mucosal immune activation [28,43]. On the other hand, these bacteria could also play a role in further increasing the risk of allergy development in FH girls, since they may contribute to increase inflammation and compromise intestinal homeostasis.

Like for non-milk consumers, the bacteria displaying altered abundances in healthy FH children were different from those altered in CMA. Again, this indicates that development of the disease is accompanied by conditions that select for specific bacteria, not already favored by the intestinal milieu present in high-risk children. In agreement, comparisons of FH children that have or have not developed CMA indicate strong microbiota alterations associated with presence of the disease (Figure 3 and Figure 4). The unstratified comparison revealed alterations in the abundances of *Intestinimonas*, Erysipelotrichaceae UCG-003, and *Anaerostipes* (Figure 3A), the genus-level groups that were altered in the comparison not taking family history into account (Figure 1A). In contrast, the abundances of these bacteria were not altered in comparisons focusing on milk consumption (Figure 2A) or presence of a family history of allergies (Figure 3A), stressing the notion that these changes are directly associated with the development of CMA. However, in the sex-stratified comparisons of FH children the decrease in *Anaerostipes* in CMA was only detected in girls whereas the increase in *Intestinimonas* was only detected in boys, again supporting different interactions between the disease and the microbiome in each sex.

Remarkably, *Anaerostipes* has been detected to be depleted in infants with CMA, and while transfer of gut microbiota from healthy infants protected germ-free mice from CMA development upon sensitization, transfer of *Anaerostipes*-depleted CMA microbiota failed to do so [9,66]. The relevance of *Anaerostipes* to the protective effect was demonstrated by the fact that its abundance in mice correlated with the expression of genes related to energy metabolism and tissue repair in intestinal epithelial cells, which could affect the intestinal environment and therefore the regulation of allergic responses to food antigens. Moreover, monocolonization with *A. caccae* reproduced the results obtained through microbiota transfer from healthy infants [9]. More recently, a synbiotic containing *A. caccae* and lactulose has been proven to both prevent and treat CMA in mouse models [18]. *Anaerostipes* may be particularly relevant to maintaining homeostasis in the gut environment as it is, not only one of the main butyrate producers of the gut, but also one of the very few that utilize lactate for butyrate production [67]. The abundance and activity of the relatively small number of lactate utilizers in the gut have been shown to be critical to the stability of the microbial community: lactate accumulation can cause severe perturbations due to its low pKa and differential effects on the growth of different microbiome species [68]. As a result, the depletion of key lactate-utilizing bacteria and the accumulation of lactate have been associated with various inflammatory gastrointestinal diseases and other negative health outcomes [68,69,70]. Our results support a critical role for *Anaerostipes* depletion in CMA development, specifically in the case of girls, perhaps due to their increased propensity to mucosal immune activation and inflammation [43].

## 5. Conclusions

In spite of the sample size reductions imposed by our stratification approach, our study uncovers sex differences in the gut microbiome that range from differential effects of having a family history of allergy or undergoing milk restriction, to different microbial alterations upon appearance of CMA, to different levels of microbiome recovery upon DOT. These results support the notion that sex-linked differences in immunity are reflected in the gut microbiome during CMA, with likely repercussions on the natural history of the disease. Our findings suggest that microbiome-targeted strategies against CMA may be more successful if specifically tailored to the alterations characteristic of each sex. Furthermore, since milk elimination from the diet produces sex-specific microbiome alterations also in healthy children, compensatory dietary or probiotic consumption strategies tailored to each sex should also be relevant for children that do not consume natural milk due to causes unrelated to CMA, such as lactose intolerance, galactosemia, or dietary preferences.

Future studies will be needed to advance beyond the different taxon correlations identified in this study and unravel the functional consequences of the sex-specific microbiome alterations in children with CMA. Metagenomic and metatranscriptomic analyses should reveal the bacterial functions that are differentially altered in boys and girls with CMA, and metabolomic measurements should identify whether microbial metabolites of relevance to gut and immune homeostasis display different concentrations in their intestinal environments. This line of research will contribute to decipher the mechanistic pathways through which bacteria exert their differential effects on the risk, development, and recovery of CMA in boys and girls, enabling the design of rational, sex-specific microbiome-targeted approaches to combat this disease.

## Figures and Tables

**Table 1:** Characteristics of participating children.

Characteristics	Healthy Controls	Cow’s Milk Allergy (CMA)	Development of Oral Tolerance (DOT)
N	36	14	18
Sex			
Female (F)	17	7	9
Male (M)	19	7	9
Milk Consumption			
Yes (MC)	26	0	13
No (NMC)	10	14	5
Family history of allergic diseases			
Yes (FH)	22	12	16
No (NFH)	14	2	2

## Data Availability

The 16S rRNA gene sequences employed in this study can be found at https://www.ebi.ac.uk/ena, accessed on 30 July 2024, under accession number PRJEB78556. Other datasets are available on request from the authors.

## Abbreviations

The following abbreviations are used in this manuscript:

CMA	cow’s milk allergy
DOT	development of oral tolerance
PERMANOVA	permutational multivariate analysis of variance
SD	standard deviation
Ig	immunoglobulin
mL	milliliter
ASV	amplicon sequence variant
ANCOM	analysis of the composition of microbiomes
log2FC	logarithm base 2 of the fold change
F	female
M	male
MC	milk consumer
NMC	non-milk consumer
FH	family history of allergic diseases
NFH	no family history of allergic diseases
DNA	deoxyribonucleic acid
rRNA	ribosomal ribonucleic acid
CCA	Canonical Correspondence Analysis
TNF-α	tumor necrosis factor Alpha
Treg cells	regulatory T cells
Th cells	T helper cells
